# Small Extracellular Vesicles in Transplant Rejection

**DOI:** 10.3390/cells10112989

**Published:** 2021-11-03

**Authors:** Justyna E. Gołębiewska, Anna Wardowska, Monika Pietrowska, Anna Wojakowska, Alicja Dębska-Ślizień

**Affiliations:** 1 Department of Nephrology, Transplantology and Internal Medicine, Medical University of Gdańsk, 80-210 Gdańsk, Poland; adeb@gumed.edu.pl; 2 Department of Physiopathology, Medical University of Gdańsk, 80-210 Gdańsk, Poland; anna.wardowska@gumed.edu.pl; 3 Centre for Translational Research and Molecular Biology of Cancer, Maria Skłodowska-Curie National Research Institute of Oncology, 44-102 Gliwice, Poland; Monika.Pietrowska@io.gliwice.pl; 4 Institute of Bioorganic Chemistry Polish Academy of Sciences, 61-704 Poznań, Poland; astasz@ibch.poznan.pl

**Keywords:** small extracellular vesicles, transplantation, biomarker

## Abstract

Small extracellular vesicles (sEV), which are released to body fluids (e.g., serum, urine) by all types of human cells, may stimulate or inhibit the innate and adaptive immune response through multiple mechanisms. Exosomes or sEV have on their surface many key receptors of immune response, including major histocompatibility complex (MHC) components, identical to their cellular origin. They also exhibit an ability to carry antigen and target leukocytes either via interaction with cell surface receptors or intracellular delivery of inflammatory mediators, receptors, enzymes, mRNAs, and noncoding RNAs. By the transfer of donor MHC antigens to recipient antigen presenting cells sEV may also contribute to T cell allorecognition and alloresponse. Here, we review the influence of sEV on the development of rejection or tolerance in the setting of solid organ and tissue allotransplantation. We also summarize and discuss potential applications of plasma and urinary sEV as biomarkers in the context of transplantation. We focus on the attempts to use sEV as a noninvasive approach to detecting allograft rejection. Preliminary studies show that both sEV total levels and a set of specific molecules included in their cargo may be an evidence of ongoing allograft rejection.

## 1. Introduction—Solid Organ and Tissue Transplantation-Potential Role of Exosomes in Monitoring and Management of Rejection

During the last decade, there has been a progressive increase in the number of solid organ transplantations. According to the Global Observatory on Donation and Transplantation (GODT) data, produced by the WHO-ONT (World Health Organization-The Spanish Transplant Organization, Organización Nacional de Trasplantes), in 2019, there were a total of circa 163,141 solid organ transplants worldwide [1]. The kidney is the most frequently transplanted organ followed by the liver and the heart. The estimated number of kidney, liver and heart transplantations worldwide in 2019 was: 105,234, 39,007 and 9140, respectively. Transplantation is a lifesaving procedure for patients with end-stage heart, lung and liver failure. For most patients with end-stage kidney disease, transplantation is the renal replacement modality of choice, providing the longest survival and the best quality of life. Pancreas and islet transplantation reduce the risk of life-threatening severe hypoglycemia in type 1 diabetics with hypoglycemia unawareness. 

Despite the use of more efficient immunosuppressive therapies and transplantation techniques, allograft rejection remains one of the main causes for allograft failure. Since infections are the principal cause of death in this population, it is quite a challenge to maintain a delicate balance between under- and over-immunosuppression in order to minimize the risk for both rejection and infectious complications. Therefore, a complex of clinical (e.g., GFR, proteinuria), immunological (e.g., donor specific antibodies DSA), instrumental (e.g., resistive index at Doppler ultrasound), and histological parameters is used to monitor solid organ allograft function. Histological examination through renal biopsy remains the gold standard for diagnosis of solid organ allograft rejection, both cellular and humoral. For that reason, surveillance biopsies allowing an early insight into an allograft status are a standard of care in heart and lung transplantation in order to enable early therapeutic intervention. However, a biopsy carries the risk of bleeding and damage to the allograft or the surrounding structures. In case of islet allotransplantation, neither biopsy nor imaging of transplanted islets are feasible, hence the only available tools to monitor islet function are c-peptide concentration and glycemia. Therefore, attempts are still being made to find a reliable, non-invasive and easy monitoring tool that could be used as both a diagnostic and prognostic biomarker.

So far, a number of potential biomarkers have been proposed in the context of acute rejection (AR). However, all have their limitations. Therefore, there is still room for improvement in the search for a perfect molecular signature of AR. Recently, attention has been given to the role of extracellular vesicles (EVs) in AR [2].

## 2. Exosomes as Multi-Faceted Organelles

Extracellular vesicles (EVs) have kept scientific attention over the past decade, due to their abundance in the human body and a broad spectrum of performed functions. The presence of these structures in the extracellular space was identified and described in 1987 by Johnstone et al. [3]. To date, research in the field of EVs has revealed the major properties of these vesicles and their great potential as diagnostic and therapeutic tools [4]. Initially EVs were reckoned as cellular waste resulting from cell damage, with no significant impact on neighboring cells [5]. Then it turned out that they act as functional vehicles responsible for the transfer of a specific cargo, i.e., proteins [6], lipids [7] and nucleic acids [6,8,9] to distinct target cells. Therefore, EVs constitute a new mode of intercellular communication with a significant impact on a variety of cellular processes and behavior [10,11,12]. The knowledge regarding EVs characteristics is constantly increasing. Nevertheless, many questions remain unanswered.

### 2.1. Origin, Characteristics and Nomenclature of Extracellular Vesicles

The recently updated guidelines of the International Society for Extracellular Vesicles (ISEV) on minimal information for studies of extracellular vesicles (MISEV) focus on the need for appropriate nomenclature of extracellular vesicles (EV) used in the research [13]. The ISEV consensus recommendation on nomenclature is to use “extracellular vesicle” as the “generic term for particles naturally released from the cell that are delimited by a lipid bilayer and cannot replicate” and to specify this term further based on their measurable characteristics such as cell of origin, molecular markers, size, density, function, etc.

EVs constitute a group of structures produced and released by almost all types of cells. They vary in size, properties and secretion depending on the cells of their origin [14,15]. The classification of EVs is constantly evolving [13], but the generic categories are: ectosomes or microvesicles, exosomes or small extracellular vesicles (in the presented work, both terms are used according to the nomenclature used in the referenced publications), and apoptotic bodies [16]. Direct outward budding of a plasma membrane is the source of ectosomes. These structures arising from a plasma membrane encompass microvesicles, microparticles, and large vesicles within the size range of ca. 50 nm to 1 µm in diameter. Contrary, sEV are of endosomal origin with their size range of ca. 40 to 160 nm in diameter [4]. They are formed in the process of inward budding in early endosomes to form multivesicular bodies (MVBs). The subsequent invagination of late endosomes’ membranes leads to the formation of intraluminal vesicles (ILVs) (future exosomes) inside larger MVBs [17]. As for apoptotic bodies (ApoBD), they are exclusively released from plasma membrane at the last stage of apoptosis. The formation of ApoBDs is an important process downstream of apoptotic cell death [18]. ApoBDs significantly vary in size and content, carrying several intracellular fragments, cellular organelles, membranes, and other cytosolic contents [19,20].

The biogenesis of sEV can be accomplished in three ways. In the case of exosomes, it can be performed through the vesicle budding into discrete endosomes that mature into multivesicular bodies which then release exosomes upon plasma membrane fusion. In the case of smallest microvesicles, it is the direct vesicle budding from the plasma membrane. Last but not least, there is the delayed release by budding at intracellular plasma membrane-connected compartments (IPMCs) followed by deconstruction of IPMC neck(s) [21]. The release of exosomes into the body fluids is mediated by various types of proteins [22,23,24] including the endosomal sorting complex required for transport–ESCRT [25]. It is a complexed machinery composed of four separate proteins (ESCRT 0-III) that cooperatively lead to MVBs formation, vesicle budding with subsequent protein cargo sorting [25]. Recent evidence shows that ESCRT is not the only exosome-releasing mechanism. An alternative pathway for sorting exosomal cargo into MVBs can be driven by proteins of the Rab-GTPase family, i.e., Rab27 or Ral [22,24,26]. Several external factors can significantly influence the mechanisms of sEV formation. Cell type, cell confluency, cell culture conditions and the presence or absence of cytokines and growth factors seem to be the most relevant in the process of in vitro sEV formation [27,28]. However, it has been confirmed that the sites of sEV production, protein sorting, physico-chemical aspects (precisely cell membrane composition including proportions of phospholipids, ceramide abundance or distribution of lipid-rafts) [21], and trans-acting mediators, can also regulate exosome biogenesis process [29]. 

The content of sEV is highly dependent on the type of cell a given vesicle originates from. That is why, sEV are often regarded as a mini version of their parental cell [30,31]. Several of the cargo molecules are involved in the biogenesis and transport capabilities of sEV. Proteins, precisely tetraspanins (CD9, CD63, CD81 and CD82) are responsible for exosomes’ cell penetration, invasion and fusion, whereas heat shock proteins (HSP70, HSP90) are involved in antigen binding and presentation [32,33]. Alix, TSG101, annexins and Rab are associated with exosome release and membrane transport and function [21,34,35]. Some of these proteins (TSG101, HSP70, CD81 and CD63), specifically enriched in exosomes, are widely used as exosomal marker proteins. Aside from proteins, sEV also contain various types of nucleic acids that can be transferred to recipient cells and modulate their function. The introduction of different types of RNAs, including: miRNAs, long non-coding RNAs (lncRNAs) or circular RNAs (circRNAs) may result in transient or persistent phenotypic changes in recipient cells [12]. Thus, exosomes’ RNAs may participate in angiogenesis, hematopoesis, exocytosis, tumorigenesis and exosome-based cell to cell communication [35,36,37]. Additionally, sEV can be released and taken up by target cells to modulate cell lipid metabolism. The following lipids are most frequently carried by sEV: phosphatidylserine (PS), phosphatidic acid, cholesterol, sphingomyelin (SM), arachidonic acid and other fatty acids, prostaglandins and leukotrienes [21]. Apart from preserving stability and structural rigidity of sEV, these lipids can play a role in lipid-related pathologies. Therefore, the lipid content of sEV can be regarded as a potential disease diagnosis and prognosis biomarker [38,39].

### 2.2. Biological Function and Role in Immunology

The internal complexity of sEV reflects their bio-functional diversity. These nanovesicles are engaged in a number of physiological processes, including intercellular signaling and cell to cell communication [40,41], cellular homeostasis [42], autophagy [43], reproduction and development [44,45,46]. These vesicles also play significant roles in human pathology, inducing or propagating different types of cancer [47], neurodegenerative diseases [48], infectious diseases [49], pregnancy complications [50] or autoimmune conditions [51]. They also participate in the allograft rejection/tolerance [52,53,54].

sEV have a double role in the immune response, since they can either stimulate or suppress the function of the immune system. On their own, exosomes do not induce any severe immune reactions, as proved in mice subjected to repeated administration of mouse or human cell-derived sEV [55]. The sEV cargo plays an important function in immunoregulation, including antigen presentation, immune activation or suppression, as well as immune tolerance via exosome-mediated intercellular communication. Various components of sEV, including nucleic acids and proteins, regulate the innate and adaptive immune response, by e.g., control of gene expression and signaling pathways in recipient cells with transfer of miRNAs [56]. These processes eventually lead to altered dendritic cell maturation. Dendritic cells (DCs), professional antigen presenting cells (APCs), load antigen-derived peptides into MHC complexes, forming pMHC (peptide-MHC) complexes that are released as exosome cargo [57,58,59]. These completely functional pMHC are available and can be picked up by different types of cells, including other APCs. Such “cross-decorated” APCs can easily display the acquired pMHC, thus presenting them to effector cells. The cross-decoration seems to amplify the immune response against a specific antigen by increasing the number of cells displaying it on their surface [60]. Interestingly, sEV with the same content (pMHC) accompanied with co-stimulatory signals, stimulate T cells less effectively compared to APCs [4]. Depending on the maturity and activation of DCs, sEV released by this immune cell subpopulation may antagonistically stimulate responding cells (i.e., T cells). Mature DCs can prime a specific activity of CD4+ and CD8+ T cells, as proven with sEV derived from DCs pulsed with a tumor peptide which led to eradication or growth suppression of established murine tumors [61]. sEV derived from DCs can also regulate immunological memory through the surface expression of MHC I and II molecules [29]. In contrast, immature DCs-derived sEV show rather modulatory function and have a tendency to suppress immune response. Such sEV are characterized with FasL expression that leads to Fas-expressing T cells apoptosis [62]. Molecules participating in cell apoptosis, e.g., FasL and TRAIL, can also be released by stimulated T cells [63]. The bidirectional molecule transfer between DCs and T cells was described and confirmed by Zhang et al. [64]. It was reported that sEV derived from T cells, both CD4+ and CD8+, could be taken up by DCs through pMHC II/TCR and ICAM-1/LFA-1 interactions. Moreover, it was demonstrated that sEV originating from CD4+ T cells and expressing FasL specifically inhibited DC-mediated CD4+ T cell stimulation. The authors of this report hypothesized that this effect may be associated with inter alia induction of apoptosis in Fas-expressing DCs via the Fas/FasL pathway, thus securing the DC-mediated T cell silence in an antigen-specific way [64]. Apart from being an effective source of sEV, T cells are extremely eager to utilize sEV of distinct origin. The sEV source and content dictate the outcome of sEV- T cell interaction. Besides well-described sEV-mediated activation of T cells, it is widely reported that the predominant result of such an interaction is T cell suppression [65]. Regulatory T cells help to maintain the immune tolerance and homeostasis through several different mechanisms, inter alia via exosomes [66]. Okoye et al. demonstrated that exosomal transfer of miRNA is central for Treg function. According to that study, Treg cells transferred Let-7d to Th1 cells thus suppressing Th1 cells proliferation and IFN-γ secretion. This silencing of Th1 cells exposed to Treg-derived sEV prevent systemic autoimmune responses [66]. The interaction between sEV released by Tregs and APCs, induces the tolerogenic phenotype in the latter, which manifests through altered cytokine secretion profile and surface expression of inhibitory molecules [67,68]. The failure of Treg-mediated T cell activation does not have to rely only on altered reactivity of APCs. Tregs can also modify adenosine production in released sEV through the activity of an immunomodulatory protein–CD73. The overproduction of adenosine results in its enhanced binding by specific receptors on CD4+ T cells, in consequence leading to cell suppression [69]. Tregs, on their own, also rely on exosomal cargo to maintain their population. The induction of CD4+CD25+FoxP3+ Tregs is partially dependent on thymic EVs that carry TGF-β [70]. B cell-derived exosomes, although significantly less explored, have already proved to be essential in immune response [60]. B cell sEV, released upon stimulation, can target T cells either to activate these cells or to induce their apoptosis. The final outcome of the mode of action of sEV, as in the case of other cell populations, depends mainly on the immunophenotype of the parental B cell [71]. 

Recently released data regarding T and B regulatory cells-associated exosomes revealed the increasing importance of one of the immunosuppressive cytokines–IL35 [72,73,74]. This potent suppressive cytokine from the IL12 family is produced by Tregs, Bregs, tissue macrophages and dendritic cells. IL35 acts through suppression of the T effector cells proliferation and by inducing the production of IL35 by conventional T cells (iTr35) [72]. The unique feature of IL35 is its ability to bind to one of the tetraspanins (CD81) present in EVs and exert its suppressory effect by interacting with IL35 receptors on another cell’s surface. According to the available data, IL35 can be either carried within exosomes released by Breg cells [73] or can coat EVs (IL35-coated EVs) produced by antigen-specific Treg cells or iTr35 cells [72].

## 3. sEV in Solid Organ Transplantation

Any type of process associated with introduction of foreign cells/tissue/organ to a recipient body induces injury and stress responses in both the transplanted structures and the recipient immunological system. The initiated rejection is the utmost obstacle in successful transplantation. The immune response triggered by the allograft can be regarded as a continuous dialogue between the elements of the innate and adaptive immune response. The innate immunity, activated shortly after transplantation, is mainly a non-specific response to tissue damage and is likely to appear regardless of genetic disparities between the donor and the recipient [75]. It seems that the transplant itself triggers reactions leading to its own destruction and subsequent rejection. The current transplant immunology dogma states that the innate immunity is not powerful enough to effectively reject an allograft on its own. However, growing evidence suggests a key role of NK cells in the pathogenesis of immune-mediated graft damage in kidney transplantation [76,77,78]. The dominating immune mechanisms associated with graft rejection are mediated by adaptive immune response elements. Two major and intertwining pathways are T cell-mediated rejection (TCMR), previously known as “cellular rejection” and antibody-mediated rejection (ABMR), formerly “humoral rejection”. Allograft rejections are also categorized according to their onset time. The first, hyper-acute reaction appears within minutes to hours post transplantation and is mediated by preformed antibodies. This fast rejection is characterized with endothelial damage and thrombosis that result in graft necrosis. The second type of rejection called acute (AR), can occur between a week and several months after transplantation. It is strictly associated with immune reaction mediated by one of two pathways, either T cell-dependent or antibody-dependent. Characteristic features of these rejection mechanisms are inflammation and parenchymal cell damage. Chronic rejection, developing within months to years after transplantation, results from generation of immune memory cells and antibodies.

### 3.1. T Cell-Mediated Rejection (TCMR) and sEV

T cell activation plays a pivotal role in graft rejection. Both CD4+ and CD8+ T cells significantly contribute to this process, but through different mechanisms. CD4+ T cells have a double task as, besides their own activation, they provide help for robust activation of donor-reactive effector CD8+ cells [79]. These effector CD8+ T cells become the major driving force of allograft destruction [79,80]. Regardless of the effector T cell subpopulation, the alloantigen presentation by dendritic cells is a critical step in the rejection process. The recognition of donor MHC molecules by T cells occurs via three distinct mechanisms: direct, indirect and semi-direct pathways [81,82].

**Figure 1:**
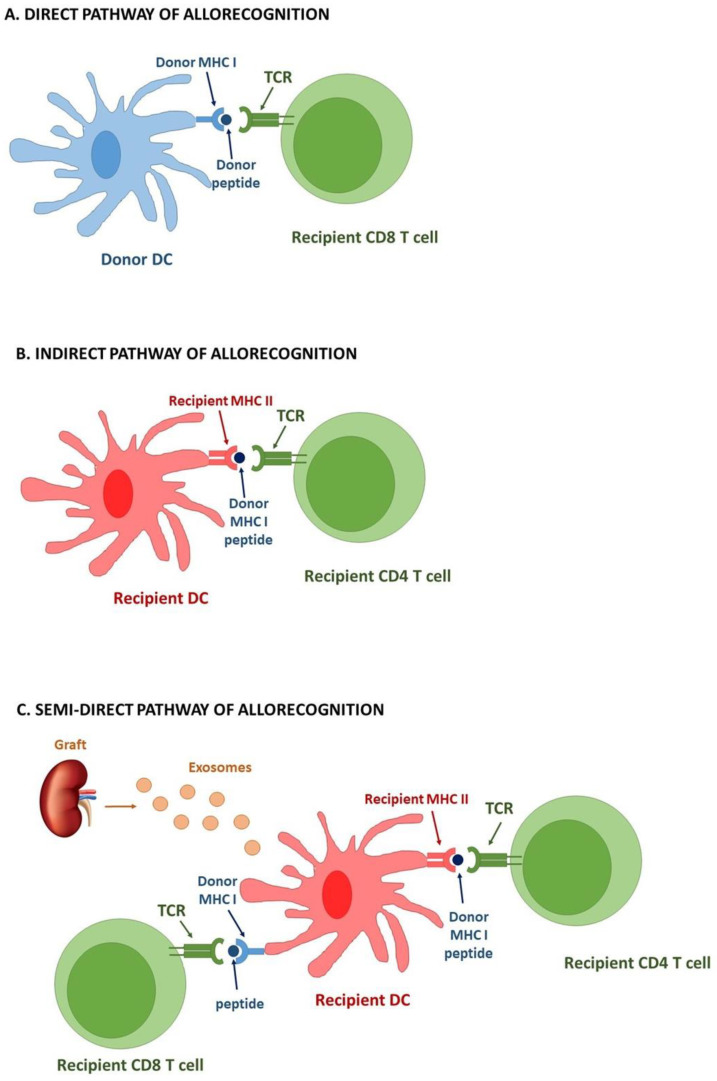
Different types of graft allorecognition mediated by recipient T cells. (**A**). Direct pathway–donor DCs migrate to recipient lymph nodes and present allo-MHC molecules to immunocompetent recipient T cells. (**B**). Indirect pathway–recipient DCs capture donor antigens (including donor MHC molecules) from the grafted tissue and via bloodstream reach recipient lymphatic organs, where appropriate presentation in the context of self-MHC to T cells takes place. (**C**). Semi-direct pathway–Donor-derived exosomes cross-decorate recipient DCs with donor intact MHC molecules that are subsequently presented to T cells.

Donor-derived DCs, one of subpopulations found among the so-called “passenger leukocytes”, are key elements of the direct pathway of alloantigen presentation (Figure 1A). These DCs present intact allogeneic MHC-peptide complexes to naïve recipient T cells in the secondary lymphoid tissue, thus providing a potent immunological stimulus for adaptive immunity [83]. The recipient-derived naïve CD4+ T cells recognize not only foreign antigens presented within MHC molecules, but also MHC complexes as discrepant and potentially dangerous agents. Such interaction between DCs and T cells requires activation of the latter via TCR receptor, cell adhesion (LFA-1/VLA4–ICAM-1/VCAM-1) and engagement of co-stimulatory molecules (CD28-B7). Since DCs express MHC I and MHC II, they can effectively prime both CD4+ and CD8+ T cells, leading to increased cytotoxicity towards the transplant, accompanied with increased IL-2-associated proliferation of T cells [84,85]. While CD8+ T cells predominantly differentiate into cytotoxic T cells, CD4+ T cells have more differentiating options, including helper T cells (Th1, Th2 and Th17) and regulatory T cell subsets. The proportions of CD4+-derived T cell subpopulations depend on the local inflammatory microenvironment [85]. Regardless of the cell subtype, graft-specific T cells can easily infiltrate the graft, where they directly recognize alloantigens and perform their graft-destructive actions. The direct pathway of allorecognition is thought to principally contribute to acute organ rejection through initiation of the adaptive immune response towards an MHC-mismatched transplant. The time-limited impact of the direct pathway on rejection mechanisms is strictly related to a finite number of donor-derived leukocytes transferred within the graft [86].

In contrast, the indirect pathway is regarded as a dominant mode of long-term allorecognition, responsible for alloantibody production and chronic graft rejection (Figure 1B) [87]. The dominance of indirect recognition of graft antigens coincides with depletion of donor APCs from the graft. At that time recipient DCs (rec-DCs) infiltrate the transplanted tissue and collect the antigens from the graft. This way recipient APCs are able to process foreign MHC molecules and present them within self-MHC to naïve immunocompetent T cells [88,89]. Moreover, at that point all proteins in the donor graft that differ from the recipients’ proteins are potential triggers of anti-graft response. Due to the different mode of alloantigen presentation in the indirect pathway, the effector mechanism also presents some disparities compared to the direct allorecognition. Presentation of graft antigens to CD8+ T cells by recipient self-MHC I, hinders the ability of cytotoxic T cells to kill parenchymal cells of the graft. It is a consequence of distinct MHC I molecules engaged in presentation in the graft and in the periphery. In order to efficiently induce T cell reaction against the transplanted tissue, DCs need to infiltrate the graft and reside in the tissue [83]. Thus, production of cytokines becomes an alternative way for allospecific T cells to damage the graft [90]. Interestingly, the donor-specific CD8+ T cells, generated through an early direct contact with alloantigens, can lead to generation of memory T cells capable of performing their action even without MHC I molecules [91]. On the other hand, the indirectly activated CD4+ T cells can provide the help of T and B cells required for alloantibody production and, therefore, participate in chronic rejection.

The last (but not least) mechanism of allorecognition is termed a semi-direct pathway (Figure 1C). The principle of T cell allorecognition is based on APCs that have acquired intact donor antigens, usually MHC molecules. The concept of surface antigen transfer between leukocytes was proposed by Lechler in 2004 and called cross-decoration [92]. Initially, cross-decoration was described as occurring through cell blebs derived from dying DCs [58]. However, the emerging research suggest that cross-decoration occurs via an exosome-mediated transfer of intact donor MHC molecules from donor APCs. Lechler’s three-cell model explains how indirect pathway CD4+ T cells cross-regulate the function of directly alloreactive CD8+ T cells on the same recipient APCs that bridge T cells engaged in the direct and indirect pathways. According to this theory, CD4+ T cells engaged in the indirect pathway interact and regulate the function of rec-DCs that acquired intact MHC molecules from donor cells. Such cross-decorated rec-DCs present unprocessed alloantigens to direct pathway T cells [92]. This mechanism is gaining growing recognition as the main driver of acute T cell activation after allotransplantation [93].

Acquisition of donor intact MHC molecules occurs via capture of clusters of donor-derived EVs that carry these MHC molecules on the vesicle surface. The donor-derived EVs are released directly by graft cells or by donor migrating DCs that reach the graft-draining secondary lymphatic organs (SLOs). This way rec-DCs, residing in the graft-draining SLOs, become cross-decorated with unprocessed donor MHC proteins. The individual rec-DCs cross-decorated with donor-derived EVs can present host MHC II molecules, loaded with donor allopeptides to CD4+ T cells through the indirect pathway. Simultaneously, the same rec-DCs present donor intact MHC molecules on the surface of EVs attached to directly alloreactive CD8+ T cells, via the semi-direct pathway. Additionally, CD4+ T cells can promote, through the indirect pathway, the maturation of MHC cross-decorated rec-DCs that subsequently stimulate the direct pathway CD8+ T cells [94]. 

The concept of “MHC-cross decoration”, coined by Yewdell and Haeryfar in 2005 [95], along with the semi-direct pathway and the three-cell model, provided the background for current understanding of the rejection process in transplantation. But in order to obtain a complete picture, new knowledge regarding the role of donor-derived exosomes had to be gained. The importance of the passenger leukocyte theory, used for many years to explain the presentation of donor MHC to recipient immunocompetent cells, has recently been confronted with new data. Two animal studies: murine heart [96,97], skin [96,97] and islet [97] transplant models showed that no or extremely few donor passenger APCs were detected in the graft-draining lymphoid organs after transplantation of fully-mismatched skin or heterotopic (abdomen) heart grafts, respectively [96,97]. Therefore, based on these results combined with the passenger leukocyte theory, no or little alloreactivity of T cells towards the graft should be elicited. Nevertheless, the immediate process of rejection is not impeded. Recent evidence [96,97] has confirmed the detection of donor MHC molecules in small clusters of EVs attached to recipient conventional DCs and B cells in the graft-draining lymphoid organs, in vascularized (heart) and nonvascularized (skin) mouse transplant models. These vesicles were identified as exosomes based on their size (76 ± 32 nm in diameter) and expression of exosome-associated surface markers–CD9 and CD63 [96]. The donor-derived exosomes remained bound to the surface of the host DCs or were internalized. The aim of the endocytosis was either further processing of alloantigens for subsequent presentation via indirect pathway, or for degradation. According to Liu et al., donor MHC molecules transferred to rec-DCs preserved their functionality which was visible in their ability to directly prime alloreactive naïve CD8+ T cells and naïve CD4+ T cells via the indirect mode [96]. In the discussed mice cardiac allograft model [96], the donor-derived red fluorescent protein (RFP)-tagged exosomes transferred to rec-DCs in secondary lymphoid organs SLOs, increased expression of the host MHC II molecules, CD40, CD80 and CD86, but not PD-L1, by the host DCs. Among factors that influence the efficacy of exosome-mediated transfer of alloantigens are the maturation status of rec-DCs, as well as contact between donor and recipient cells. It was described by several studies, that mature DCs transferred more MHC molecules compared to immature DCs [92,96,98,99]. Moreover, this transfer can be severely affected when donor and host cells are separated by 0.4-μm-pore transwells. This finding may indicate that contact between donor and acceptor cells is necessary for release or intercellular transfer of EVs. Liu et al. confirmed this hypothesis in their animal heart transplantation model, revealing that clusters of donor-derived EVs were released into the gap between donor and acceptor DCs that were in close apposition [96]. Additionally, to support the role of rec-DCs cross-dressed with donors’ MHC as triggers of T-cell allorecognition in SLO, Liu and colleagues depleted rec-DCs in vivo. Such intervention resulted in decreased presentation of donor intact MHC molecules to directly alloreactive T cells via the semi-direct pathway, and significantly delayed acute cardiac allograft rejection in mice [96].

Marino et al. [97] suggested that donor exosomes could be released by the passenger leukocytes mobilized to graft-draining SLOs, donor cells within the graft, or both. This statement was a response to the observation of relatively few donor passenger leukocytes reaching graft-draining SLOs within the first week after heart or pancreas transplantation in mice allograft models [96,97]. The first week post transplantation is regarded critical in terms of T cell allosensitization. In the case of non-vascularized skin allografts, or other graphs that hinder donor leukocytes migration out of the graft, donor-derived exosomes leak in the graft bed passively via the severed openings of recipient lymphatic capillaries towards the graft-draining lymphoid organs. Endothelial, parenchymal or non-migratory stromal cells from the allograft are the collateral source of donor-derived EVs. It also turned out that exosomes were efficiently carried across the lymphatic endothelium in vitro, and were rapidly transported within lymphatic vessels in vivo [100]. By bearing specific glycoproteins decorated with sialic acids, exosomes enhance their recognition by macrophages in subcapsular sinus of lymph nodes thus facilitating their transfer to lymphatic system. Moreover, donor-derived exosomes can stimulate lymphatic vessel formation themselves [101]. MHC-cross decoration of rec-DCs also takes place in a graft itself.

### 3.2. Antibody-Mediated Rejection (ABMR) and sEV

Antibody-mediated rejection (ABMR), collectively with TCMR, can take part in graft rejection at every stage of this process. In the hyper acute rejections, pre-existing antibodies become the primary trigger of endothelial damage and thrombosis. Inflammation and parenchymal damage visible in acute rejection are not only T cell-dependent processes, but are also associated with specific antibodies production. Acute and chronic AMBR can be triggered by memory cells in sensitized recipients, or by de novo generated DSA in nonsensitized ones [102]. It has been implicated that ABMR is the leading cause of late allograft loss [103,104]. 

B cells may participate in graft recognition and rejection in two ways, either in cellular or in humoral reactions. In cell-mediated reactions, B cells primarily act as antigen presenting cells, thus leading to T cell activation. Alloantigen’s fragments, presented by MHC II molecules expressed on B cells, directly interact with primed Th2 cells in the indirect pathway of antigen presentation [105]. The accompanying presence of co-stimulatory signals (i.e., CD28, LFA1, CD2), adhesion molecules (i.e., B7, ICAM, LFA3) and cytokines (IL-2, IL-4, IL-6, IL-12, TNFα, IFNγ), results in activated B cell division and differentiation [106,107]. Some of these cells amplify the immune response by migration to lymph nodes, where an interaction between B cells and T cells significantly increases. Interestingly, mature plasma cells are able to produce antibodies in T cell-independent manner. The generated memory B cells additionally facilitate and exacerbate ongoing episodes of rejection [108].

DSAs, produced by activated B cells are considered a major reason of AMBR [107]. Furthermore, non-HLA antibodies, including endothelial or xenogenic antigens, as well as incompatible ABO blood group antigens, have been associated with AMBR and graft loss. Therefore, the presence of these antibodies, along with anti-HLA Abs is considered a contraindication to transplantation [109]. De novo generation of DSAs is a consequence of naïve B cell activation following alloantigen recognition via B cell receptor (BCR). Alloreactive B cells can either differentiate into short-lived (extrafollicular) plasma cells or contribute to memory compartment residing in germinal centers (GC) of lymph nodes [110]. Extrafollicular plasmablasts are the source of low-affinity IgM antibodies. In contrast, GC-memory B cells undergo somatic hypermutation, class switching and affinity maturation upon interaction with follicular DCs and follicular helper T cells [111]. 

**Figure 2:**
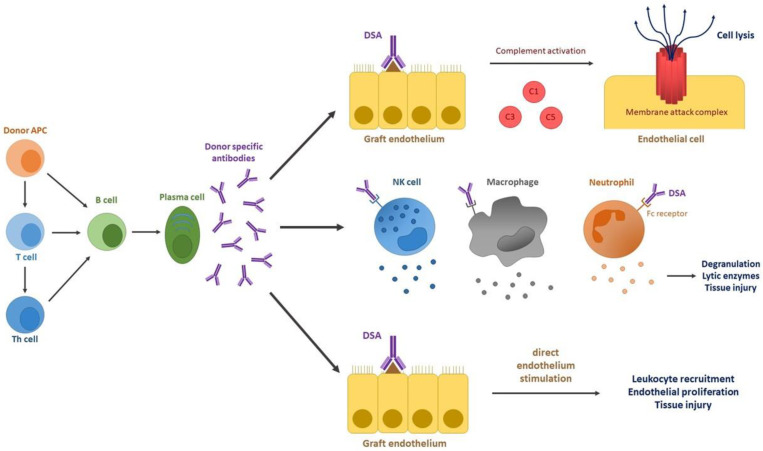
Donor specific antibody-related mechanism leading to graft loss in antibody-mediated rejection. DSAs lead to tissue damage through three mechanisms, dependent on and independent of complement biding. The first mechanism relies on activation of the complement classical pathway activation. Formation of the membrane attack complex (MAC) results in graft endothelial cell lysis. The second mechanism engages the innate immunity cells in antibody-dependent cellular cytotoxicity. Upon FC receptor stimulation with DSAs, NK cells, monocytes, and neutrophils release lytic enzymes, thus leading to tissue damage. In the last mechanism, direct interaction of DSAs with a graft tissue results in excessive activation of graft endothelium, leukocyte recruitment and subsequent tissue injury. APC-antigen presenting cell; Th–T helper; C1, C3, C5–complement proteins.

There are three major mechanisms through which DSAs induce reactivity towards the grafted tissue (Figure 2). Firstly, the interaction between DSAs and their target antigens on graft endothelial cells causes activation of the classic complement pathway. This key pathological process of acute AMBR relies on binding of C1q to the Fc fragments of bound DSAs with subsequent cascade of enzymes, membrane attack complex (MAC) formation and cell lysis [80]. Apart from direct initiation of tissue damage through MAC, the complement system can augment alloreactivity via additional mechanisms. The release of anaphylatoxins (C3a, C5a) induces the innate immunity leading to increased vascular permeability [112]. Other proteins from the complement cascade can promote phagocytosis or activate production of pro-inflammatory cytokines by endothelial cells. Additionally, augmentation of T cell-mediated rejection may result from an interaction of the complement with the adaptive immunity [85]. In the absence of the complement activation, the second mechanism of DSA-associated reactivity can be launched. The antibody-dependent cellular cytotoxicity is based on innate immune cells (including neutrophils, macrophages and NK cells) binding to Fc fragments of DSAs, followed by cell degranulation and release of lytic enzymes. This process, resulting in tissue injury and endothelial cell death, is proposed as important pathogenesis in subclinical and chronic ABMR [113,114,115]. The third mechanism of DSAs-associated graft injury is the direct activation of endothelial cells. The DSAs ligation on endothelial cells activates intracellular signaling cascades that lead to a reaction similar to transplant vasculopathy (TV). TV is a vasculo-occlusive disease resulting in ischemic injury and deterioration of organ function. Endothelial cells, in response to DSAs ligation induce proliferation and upregulate expression of several growth factor receptors, including FGFR or leukocyte adhesion molecules, i.e., VCAM-1. This perpetuates microvasculature injury through excessive proliferation of endothelial cells and recruitment of activated effector cells damaging the graft [116,117]. The first, complement-dependent mechanism of DSAs mode of action is strictly associated with the C4d deposition in allograft, whereas the latter two complement-independent mechanisms can explain the clinical phenotypes of antibody-mediated rejection with negative C4d staining in peritubular capillaries. 

Even though B cells were confirmed as sEV producers in the late 1990s [118,119], little has been done to elucidate the role of B cell EVs in immunology. The currently available data regarding B cell sEV comprise few articles describing their role in a limited number of immunological contexts. Although several recently published manuscripts rise the issue of exosomes in ABMR, none of them focus precisely on exosomes produced by B cells. The majority of these papers utilize human plasma or urine exosomes without identification of the source of exosomes [54,120,121]. Nevertheless, a study by Raposo et al. performed in 1996 paved the way for further discovery of B cell-associated sEV and their role in graft allorecognition. The mentioned study, performed on human and murine B cell lines, revealed B cell sEV as transporters of MHC II and associated antigens that could further mediate antigen presentation [118]. Two years later, the same laboratory reported expression of co-stimulatory molecules (CD86) and tetraspanins (CD37, CD53, CD63, CD81, and CD82) on sEV released by human B lymphocytes, thus suggesting the immunological role of B cell sEV in vivo [119]. Later it was proved that sEV release was not a constitutive activity of B cells, but required appropriate cell signaling. Upon stimulation via CD40 and IL-4, murine splenic B cells released high levels of sEV, expressing MHC I, MHC II, CD45RA, components of the BCR complex and tetraspanins [122]. Buschow et al. meticulously analyzed the content of human B cell exosomes indicating the presence of a small subsets of proteins, including HSC71, HSP90, 14-3-3e, CD20 and pyruvate kinase type M2 (PKM2) [123]. This way they significantly contributed to elucidation of exosomes’ biogenesis and function. More recent data provided a new insight into the biological activity of B cell sEV with the focus on exosome-mediated CTL immunity in vivo. The study in mice revealed that exosomes released by activated B cells can be acquired by CD8α+ DCs which subsequently prime CTL responses with the help of CD4+ T cells and NK cells [124]. Human B cell exosomes can directly target T cells by delivery of specific antigens (i.e., allergens), thereby inducing T cell proliferation accompanied with Th2 cytokine synthesis (i.e., IL5, IL-13) [125]. The Lundy’s laboratory reported the same exosomes exerting the adversative effect on immunocompetent T cells that was observed as increased apoptosis of T cells [71,126]. Human activated B cells exhibited a high capability to constitutively produce MHCII+FasL+ sEV that induced apoptosis in CD4+ T cells. Interestingly, the analyzed B cells showed high intracellular expression of FasL accompanied with no detectable FasL expression on the cell surface. This mechanism is hypothesized to be responsible for immunosuppressive activity of B cell-derived sEV. Therefore, it can be stated that the final function of B cell exosomes is strictly associated with immunophenotype of the parental B cell.

### 3.3. sEV in Allograft Tolerance Induction

Immunological tolerance is the absence of immune response toward specific antigens, but with preserved immune reactivity to others. This state is achieved through selective suppression of the immune reaction in specific settings. There are several ways through which the tolerance state can be induced. Central tolerance, generated in the immature embryonic stage of the immune system, provides non-response tolerance to the body’s own autoantigens. Therefore, it protects from unwanted reactivity of the immune system towards own tissues. According to the clonal selection theory, coined by Burnett as a consequence of the findings of Owens and Medawar, during the early development stage of the immune system a body can become tolerant to foreign antigens because of reactive lymphocyte clearance [127,128,129]. An exposure to alloantigens at the embryonic stage of development, called chimerism [130], could promote graft survival. Several mice models of chimerism were established to achieve immunological tolerance, but the majority of them resulted in a strong Graft-vs-Host Disease (GvHD) [131,132,133]. 

The immune homeostasis of a human body is also ensured by peripheral tolerance mechanisms that include clonal clearance, immunological ignorance, clonal anergy, immunological privilege, and the function of immunoregulatory cells [134]. In transplantation tolerance the anergy or depletion of alloreactive T cells provides the most profitable outcome. Nevertheless, an effective immunoregulatory activity of regulatory cell populations is desired, as the role of regulatory T or B cells is gradually getting more recognition. Regulatory T cells exert their immunomodulatory mechanisms through suppressive cytokine milieu (IL-10, TGFβ, IL-34), induction of cytolysis, interference of targeted cell metabolism and modulation of DCs activity. Tregs are one of the best explored and characterized regulatory cell subsets whose function and application have been meticulously reviewed elsewhere [135,136,137]. Regulatory B cells (Bregs), identified based on the increased production of IL-10, are a less studied regulatory cell population. Even though, Bregs are described as cells suppressing inflammatory responses in autoimmune disease, allergy, infection, transplantation, and cancer; there are several subpopulations with discrepant capabilities (reviewed in [138,139]). There is a subset of B cells expressing FasL, named “killer B cells” by Lundy’s team, which induce apoptosis of alloreactive CD4+ T cells. Interestingly, this subpopulation of killer B cells is able to release FasL with MHC II via exosomes thus resulting in target T cell death [71,126].

Exosomes are multipotent structures exerting diverse effects on target cells. In transplant immunology they act as a double-edged sword, either contributing to allorecognition and graft rejection or preventing the graft loss via induction of a suppressive phenotype of immune cells. Exosome-dependent allograft tolerance can be induced through several complex mechanisms, including split-tolerance and MHC cross-decoration. The phenomenon of split-tolerance and occurrence of non-inherited maternal antigens (NIMAs) is associated with maternal microchimerism (MMc). During pregnancy and breastfeeding a small number of maternal immune cells transferred to the offspring evokes tolerance to NIMAs in the latter [140,141]. MMc is driven by sEV, efficiently produced and released by maternal immune cells. NIMAs in maternal sEV cross-decorate host (offspring) DCs which in response upregulates endogenous PD-L1 expression and induces NIMA-specific T cell clonal anergy [52]. The PD-L1-dependent abortive proliferation of alloresponsive CD4+ T cells was mediated via the indirect pathway of allorecognition. Therefore, the split-tolerance phenomenon silences only the indirect pathway, while preserving functional direct allorecognition. Consequently, even with the increase in episodes of acute rejection in the presence of NIMA-containing EVs, a chronic metastable allotolerance can be developed [142,143]. Animal studies in NIMAs murine model confirmed anticipation of MMc-derived sEV in semi-direct pathway of T cell allorecognition, thus providing a physiological link between microchimerism and split-tolerance. It was found that while interaction of host DCs with MMc-derived sEV via the indirect pathway resulted in effector T cell anergy, semi-direct allorecognition primed immunocompetent cells [52]. The phenomenon behind chimerism-induced allotolerance in transplantation, first observed by Owen and Medawar, was clarified with the discovery of exosomes. The situation proved analogous to maternal chimerism and NIMAs. Post transplantation donor passenger DCs release sEV, capable of cross-decoration of rec-DCs, which results in CD8+ T cell activation via the semi-direct pathway. Simultaneously, the cargo of donor DC-derived sEV undergoes endocytosis by recipient APCs and is processed for subsequent presentation in self-MHC II. This indirect activation of CD4+ T cells provides cytokine support of direct CD8+ T cell priming. Additionally, the presence of CD86 on the surface of sEV facilities T cell priming through both pathways of allorecognition. On the other hand, tolerogenic mi-RNAs present in certain types of sEV, irrespective of CD86 presence, may induce anergy in CD4+ T cells, thus reducing CD8+ T cell involvement in injurious allograft recognition [52]. 

Thymocyte release of sEV can influence the tolerance state through development and proliferation of T reg cells responding to TGFβ carried in EVs [70]. Exosomes from bone marrow-derived DCs may also participate in tolerance induction utilizing MHC, as proved in a rat heart transplant model [144]. These observations confirmed the potential of sEV as effective carriers of various molecules and led to implementation of exosomes as a method of delivery of immunosuppressive agents in allograft tolerance induction [145]. Moreover, modification of exosomes cargo may exert a beneficial effect on a transplanted tissue. Ma et al. [146] proved that exosomes derived from immature DCs and donor Tregs effectively induced tolerance in a rat liver transplant model. Enhancement of immature DCs with specific molecules i.e., Fas-L or IL-4 also resulted in increased graft tolerance [146]. miRNA, abundant in physiological exosomes, seems to be an attractive molecule in transplant tolerance acquisition. A study on xenoislet transplant in humanized mouse model described the impact of donor-derived exosomal miRNAs on the graft model. The authors demonstrated that donor xenograft sEV released several miRNAs into the recipient’s circulatory system [147]. One of the miRNAs detected in the transplant islet-specific exosomes [147], namely: miR-122 has already been proven to exert its immunosuppressive potential in corneal transplantation [148]. Additionally, an altered miRNA profile of islet sEV was observed in the state of immune rejection, thus suggesting the role of exosomal miRNAs not only in modulation of post-transplant immune response but also their potential usefulness as biomarkers of the transplantation status [147].

## 4. Liquid Biopsy with the Use of sEV in Solid Organ and Tissue Transplantation

The term “liquid biopsy” applies to the sampling and analysis of non-solid biological tissue, primarily blood. This type of analysis originally referred to diagnosis of solid tumors by means of harvesting and analyzing circulating tumor cells. This term was first proposed in 2009, during the Seventh International Symposium on Minimal Residual Cancer (ISMRC) in Athens, Greece. 

Later it was implemented in other clinical fields, such as transplantation. It was established that a graft can release exosomes into the bloodstream of a recipient. So far, exosomes have been studied in the context of heart, lung, kidney and pancreatic islets transplantations. Vallabhajosyula et al. were able to show specificity and reliable characterization of both pancreatic islets exosomes and kidney allograft donor exosomes for follow-up periods of five years [147]. Sharma et al. showed that exosomes isolated from lung, heart and kidney transplant recipients with chronic allograft dysfunction had increased expression of tissue-associated self-antigens [149]. Findings of pre-clinical studies on the use of exosomes as a biomarker for various clinical conditions in solid organ transplantation are summarized in Table 1.

A special attention has been given to miRNA as a potential biomarker of the transplanted organ status. miRNAs are one of the most important types of non-coding RNAs contained in sEV. These short (ca.22 nucleotide) non-coding RNAs are known as molecules able to repress and degrade target mRNAs, thus silencing gene expression [178]. miRNAs originate from polymerase II (Pol II)–synthesized, mRNA-like primary transcripts subsequently processed by the nuclear ribonuclease Drosha. Pre-miRNAs are then exported to the cytoplasm, where they undergo further processing performed by the endoribonuclease Dicer. Dicer-dependent cleavage creates a mature miRNA that can be subsequently loaded into the RISC complex and bind with its target mRNA [179]. Exosomal miRNAs play an essential role in regulating cross-talk between cells, which proves important in health and disease. In most cases, these miRNAs exert regulatory function through interaction with mRNAs leading to alteration in gene expression [180]. However, because of resistance to RNase-dependent degradation, exosomal miRNAs have caught scientists attention as diagnostic biomarkers [181]. Up to date, the most studied scientific area of exosome miRNAs is cancer [180], where these molecules are regarded as potential predictors of tumor response to treatment, tumor biomarkers or therapeutic factors [182]. Based on the emerging data from other scientific fields, it is expected that exosomal miRNA profiling could be a non-invasive method to detect disease or a way to monitor progression or treatment efficacy. miRNA profiling has been employed in a small number of studies in heart [159] or kidney transplant recipients [169,171,174,175]. Proposed signatures included several miRNA species overall, while none recurred in different signatures. Detailed findings of these studies are provided in Table 1 and in the text below. 

### 4.1. Lung Transplantation

Exosomes have been extensively studied as a biomarker for transplanted lung rejection by Mohanakumar and colleagues. Gunasekaran et al. [150] analyzed exosomes from serum and bronchoalveolar lavage fluid (BAL) from 30 lung transplant recipients who were stable, had AR, or had bronchiolitis obliterans syndrome (BOS). Unlike stable lung transplant recipients, exosomes from both serum and BAL of recipients with acute rejection or BOS, contained donor HLA, lung-associated self-antigens (SAgs) and a specific set of miRNAs. Exosomes carrying SAg, collagen V, were found in the serum of recipients 3 months before AR and 6 months before BOS diagnosis [150]. In another study exosomes isolated from sera of 10 lung transplant recipients with bronchiolitis obliterans syndrome contained increased levels of lung SAg (Col-V and Ka1T) and MHC-II molecules, unlike exosomes found in sera of stable lung transplant recipients. Exosomes from patients with diagnosed BOS, induced immune response in mice with antibody against human HLA class I, and lung SAg (Col-V and Ka1T) production and significantly increased levels of cytokines such as IL-2, TNF-α; they also decreased IL-10 level in comparison to mice immunized with stable LTxR exosomes. Exosomes isolated from both serum and BAL from patients with BOS contained increased SAgs, natural killer cells markers CD56 and NKG2D, as well as cytotoxic molecules perforin, FasL [157]. In a study published in 2019, performed for a group of 90 lung transplant recipients with various clinical conditions including: primary graft dysfunction, respiratory viral infection, acute rejection, de novo formation of donor specific antibodies or bronchiolitis obliterans syndrome and recipients with stable lung allograft function, exosomes isolated from all of the aforementioned post-LTx clinical conditions contained important lung SAgs, immunoregulatory molecules and stress markers, which were notably absent in exosomes isolated from stable patients. Moreover, exosomes isolated from patients with symptomatic RVI did not include costimulatory molecules CD80 and CD86 in their cargo, contrary to other aforementioned pathologies [152]. These results were consistent when validated in external populations of lung transplant recipients [150] or pediatric patients [155]. Later, Gunasekaran et al. showed that in lung transplant recipients with respiratory viral infections exosomes contained also viral antigens and elicited immune response that resulted in the development of chronic lung allograft dysfunction in immunized mice [156]. In a lung transplant recipient with COVID-19, the SARS-CoV-2 spike protein was found in exosomal cargo. After resolution of infectious symptoms, exosomes with SARS-CoV-2 spike protein were no longer detected; however, exosomes with lung self-antigens and HLA class II molecules persisted, along with a progressive decline in spirometric flows, suggesting chronic lung allograft dysfunction [158]. Application of liquid chromatography−tandem mass spectrometry to profile exosome cargo from 90 lung transplant recipients enabled Bansal et al. to identify unique signatures corresponding with AR, BOS and RVI with significantly deregulated proteins in each condition [153].

### 4.2. Heart Transplantation

In 2017 Sukma Dewi et al. published a paper describing mi-RNA profiling in a group of 5 heart transplant (HTx) recipients with diagnosed acute rejection and 5 stable HTx recipients, and showed that exosomal miR-142-3p increased during rejection and augmented vascular permeability through down-regulation of endothelial RAB11FIP2 expression [159]. Kennel at al. analyzed sera of healthy individuals, patients with heart failure, HTx recipients with no rejection, TCMT or ABMR using liquid chromatography–tandem mass spectrometry (LC-MS/MS). They found clustering of three groups using principal component analysis (PCA): (1) healthy controls and heart failure patients; (2) heart recipients without rejection; and (3) heart recipients with TCMR and ABMR. They also identified a protein signature of 15 proteins that distinguished between HTx recipients without rejection and those with TCMR or ABMR [160]. In the case of humoral cardiac allograft rejection, C4d protein was present in the donor heart exosomes subset, which resolved with treatment [161]. In a study by Castellani et al. exosomes from 90 cardiac allograft recipients were analyzed with the use of surface marker analysis by multiplex flow cytometry. A total 53 cases were employed as a training cohort and 57 as a validation cohort. Concentration of exosomes was significantly increased and their diameter decreased in both TCMR and ABMR. Among EV surface markers, CD3, CD2, ROR1, SSEA-4, human leukocyte antigen (HLA)-I, and CD41b discriminated between controls and patients with TCMR, whereas HLA-II, CD326, CD19, CD25, CD20, ROR1, SSEA-4, HLA-I and CD41b discriminated controls from recipients with ABMR [162]. All authors concluded that circulating EVs are very promising as a new tool to characterize cardiac allograft rejection, even though the results were inconsistent due to different methodology applied.

### 4.3. Kidney Transplantation

In the context of KTx, studies have investigated RNA cargo and the proteome of exosomes from urine and plasma in search for potential biomarkers of acute rejection, both cellular and humoral, delayed graft function, interstitial fibrosis and tubular atrophy, calcineurin inhibitors’ (CNI) toxicity and non-specific kidney allograft dysfunction. In one of the first studies investigating urine exosomes in KTx recipients, the authors hypothesized that determination of NGAL in urinary exosomes would be a better predictor of kidney dysfunction after KTx than other urinary fractions [163]. Indeed, exosomes expressed higher levels of NGAL than the cellular fraction, and neutrophil gelatinase-associated lipocalin (NGAL) expression in exosomes was elevated in patients with delayed graft function (DGF) compared with non-DGF patients, while there were no significant differences in NGAL expression in the cellular fraction of urine [163]. In another study by Peake et al. urine protein levels of NGAL and IL-18 corresponded with day 7 post-transplant creatinine reduction ratios, unlike urine exosomal mRNA for these proteins [165]. Wang et al. [169] used high throughput sequencing of the miRNA profile of exosomes in the peripheral blood of kidney transplant recipients to diagnose DGF, defined as a dialysis requirement during the first week after renal transplantation. They found 52 known and 5 conserved exosomal miRNAs specifically expressed in recipients with DGF. Three co-expressed miRNAs, hsa-miR-33a-5p_R-1, hsa-miR-98-5p, and hsa-miR-151a-5p, were highly upregulated in the peripheral blood of KTx recipients with DGF [169]. There have also been attempts to use exosome profiling to diagnose AR. In a group of 213 KTx recipients and 14 kidney and pancreas recipients the total load of serum exosomes increased in patients with AR, whereas in patients with no rejection the levels of circulating EVs did not change. After a treatment for rejection, circulating exosomes were rapidly decreased in patients with negative peritubular capillary (PTC) C4d, but decrease was slower in patients with positive PTC C4d [164]. Sigdel et al. identified a total of 1018 proteins in unfractionated whole urine and 349 proteins in urinary exosomes. In patients with AR 11, proteins involved in inflammatory and stress response were more abundant in urinary exosomes of recipients with AR than in KTx recipients with stable allograft function [166]. In a study by Lim et al. expression of 46 out of 169 identified urinary exosomal proteins was increased in stable KTx recipients, while 17 proteins had increased expression in kidney transplant recipients undergoing acute T cell-mediated rejection (TCMR). Two proteins, tetraspanin-1 and hemopexin were significantly higher in TCMR patients [53]. Tower et al. described a significant increase in the plasma density of C4d+/CD144+ microvesicles (an endothelial marker) in KTx recipients with AMR compared to those with no AMR or healthy subjects. C4d+/CD144+ microvesicle concentration decreased by 72% in nine patients who underwent treatment for acute AMR [168]. In another study the authors identified 4 genes (gp130, CCL4, TNFα, SH2D1B, CAV1) in plasma exosomal RNA whose mRNA transcripts were significantly elevated in cases of kidney allograft antibody mediated rejection (AMR) in comparison to patients with cell-mediated rejection and control groups with no rejection [54]. Yang et al. reported no significant differences in CD4+CXCR5+ and CD4+CXCR5+CXCR3+CCR6-exosomes between AMR and non-AMR groups, whereas the proportion of CD4+CXCR5+CXCR3-exosomes was significantly higher in the AMR group when compared to the non-AMR group [170]. Park et al. proposed a urine-based platform to detect kidney transplant rejection termed iKEA (integrated kidney exosome analysis). With the use of this platform the authors found a significantly higher level of CD3+ exosomes among patients undergoing cellular rejection, very low CD3+ exosomes levels in BKV (BK virus) nephropathy and chronic antibody-mediated rejection patients [167]. In a recently published study by El-Fekih et al. an analysis of 192 urine samples and matching biopsy specimens from 175 KTx recipients undergoing for cause biopsy showed that an exosomal mRNA signature discriminated between biopsy samples from patients with all-cause rejection and those with no rejection, and an additional gene signature discriminated patients with ACR from those with AMR [121]. In a study by Takada et al. SYT17 proteins were detectable in urinary exosomal fractions with significant increase of SYT17 level in exosomes from urine of patients with chronic active AMR compared to healthy volunteers and individuals. Interestingly, no SYT17 protein was detected using whole-urine samples [120]. Changes in exosomal cargo corresponding with IF/TA on kidney allograft biopsy were also reported with either higher expression of miR-21, miR-142-3p and miR-221 [171] or higher expression of vitronectin [172]. In another study analyzing urinary exosomes, three overexpressed urinary exo-miRs (miR-146b, miR-155, andmiR-200a) in kidney recipients were negatively correlated with TAC dose, while miR-200a was positively correlated with proteinuria [174]. Investigators were also able to identify significant changes in exosomal cargo corresponding with calcineurin inhibitors toxicity [173] or eGFR < 60 mL/min/1.73 m^2^ [175]. Candidate biomarkers for different clinical conditions were summarized in Table 1.

### 4.4. Liver Transplantation

So far, only a single study investigated exosomes in order to identify rejection and predict the outcome of liver transplantation. The authors analyzed the presence of galectin-9 protein in liver exosome cargoes from three sets of paired patients, with and without acute cellular rejection and in liver samples from 73 liver transplant recipients with rejection and 63 recipients with normal liver allograft function. Expression of galectin-9 was significantly higher in the TCMR group than in the non-rejection group, both in circulating exosomes and in liver samples. The recipients with higher liver expression of galectin-9 had worse survival [177].

### 4.5. Pancreatic Islets Transplantation

Exosomes have also been investigated as biomarkers of pancreatic islet allograft rejection in a human-to-mouse xenogeneic pancreatic islet transplant model. The authors isolated islet transplant exosomes from the recipients’ blood using an anti-HLA antibody. A significant decrease in the transplant islet exosome load and changes in the exosomal miRNA and proteomic profiles were detected prior to the appearance of hyperglycemia [147]. During the entire rejection process the recipients’ T cell exosome signal was also elevated. Next, the authors used the same approach in a clinical setting to monitor exosomes in five pancreatic islet transplant recipients [176]. Plasma samples were collected at baseline and for up to five years post-transplant. By analyzing transplant islet exosome load and changes in the exosomal miRNA it was possible to identify ongoing destruction of transplanted islets before the onset of hyperglycemia, and to differentiate between the recurrence of autoimmunity and alloimmunity. In 4 out of 5 type 1 diabetics the islet exosome signal remained stable throughout the whole observation period, while in one patient a significant drop in islet exosome quantity preceded the onset of hyperglycemia. Further analysis showed decrease in insulin-containing exosomes, but not glucagon containing exosomes, indicating selective destruction of β-cells typical for autoimmune process.

## 5. Conclusions

Over the past few years, sEV including exosomes have attracted great attention as a potential tool for examining transplanted organ/tissue physiology and disease. The utility of sEV has been investigated across various transplanted organs and various medical conditions including AR, using diverse biological samples, with focus on different elements of sEV cargo and with the use of several distinct techniques. In the case of lung transplantation EVs have been most extensively studied by Mohanakumar and colleagues who identified sets of surface molecules specific for BOS, AR and viral infections with the use of Western Blot technique. The researchers verified their results in small external populations, including pediatric patients. In heart transplantation, either miRNA or proteomic molecular signatures of AR were proposed based on sEV profiling. Neither of these were validated in external cohorts. In kidney transplantation, a number of potential molecular signatures of the most important both immunological and non-immunological causes of early and late graft dysfunction have been proposed. However, all were studied in small groups of cases, the results were inconsistent and none were validated in multiple independent cohorts. Only single studies have investigated sEV in the case of liver and pancreatic islets transplantation. Even though the published data seems promising, future studies are required before sEV analysis could facilitate clinical decision-making.

Research on practical application of sEV is much hampered by an experimental setup where they form a heterogeneous population of vesicles released by various types of cells. This is the case, inter alia, in serum of patients after transplantation, where sEV released by donor’s cells are present alongside sEV produced by recipient’s cells. Small extracellular vesicles of lymphoid origin represent the largest group among all sEV identified in serum or plasma, which is a result of a large number of lymphocytes circulating in blood. It is estimated that sEV released by lymphocytes represent 60–80% of exosome population in serum/plasma, which poses an obvious problem when one aims at analyzing the composition of exosomes to be isolated from the general population of sEV. Furthermore, no standardized procedures for isolation of different EV types exist, leading to differences in contamination levels and co-isolation of various vesicles. The criteria and methods of EV characterization are also unclear and seem to change as we progress in understanding heterogeneity of EV. However, constantly improved sensitivity of instrumentation dedicated to analysis of sEV gives hope. Modern technologies enabling fast and sensitive detection of antigens present on the surface of sEV offer new opportunities of their monitoring. Nevertheless, future validation studies encompassing all aspects of sEV isolation, characterization, and signaling seem to be necessary to move the field forward and translate the current knowledge to clinically applicable strategies and methods. In this respect, antibody-based microarrays, multiparameter quantitative flow cytometry, and high resolution mass spectrometry-based proteomics are emerging as tools applicable to serial monitoring of sEV in body fluids of transplant recipients.

## Figures and Tables

**Table 1:** Findings of pre-clinical studies on the use of exosomes as a biomarker for various clinical conditions in solid organ or tissue transplantation.

Study First Author, Year	Group	Sample Type and Method of Analysis	Results
Sharma, 2018 [149]	30 LTxRs 10 with BOS, and 10 in stable condition, 5 patients developed DSA and 5 without DSA considered as stable 8 HTxRs 5 CAV; 3 stable with no evidence of rejection. 15 KTxRs 9 had biopsy-proven TG 6 had a biopsy that confirmed the absence of rejection pathology and a well-functioning kidney	Sample type: serum Analysis: Western blot, using antibodies to CD9 exosome-specific markers. Tissue-associated lung SAgs, collagen V (Col-V) and K-alpha 1 tubulin (Kα1T), heart SAgs, myosin and vimentin, kidney SAgs, fibronectin and collagen IV (Col-IV).	LTxRs with BOS had exosomes with higher expression of Col-V (4.2-fold) and Kα1T (37.1-fold) than stable LTxRs HTxRs with CAV had exosomes with a 3.9-fold increase in myosin and a 4.7- fold increase in vimentin compared with stable HTxRs. KTxRs with transplant glomerulopathy had circulating exosomes with a 2-fold increased expression of fibronectin and 2.5-fold increase in Col-IV compared with stable KTxRs.
**Lung**			
Gunasekaran, 2017 [150]	30 bilateral LTxRs 10 with AR 10 LTxRs diagnosed with BOS, and 10 stable LTxRs	Sample type: serum, BAL fluid collected at 1, 3, 6 and 12 months after LTx and as required for clinical diagnosis Analysis: Western blot, using antibodies to exosome-specific marker annexin V and for lung-associated SAg Col-V, Flow cytometry for exosome surface marker analysis (CD63, anti-HLA A2 anti-HLA A3–to differentiate donor and recipient exosomes) miRNA profiling, TaqMan miRNA real-time polymerase chain reaction	Exosomes containing SAg Col-V are induced by alloimmune responses after human LTx SAgs Col-V and Ka1T were present on the surface of exosomes isolated from LTxRs with BOS and AR containing the SAg Col-V were demonstrable in the sera of LTxRs with AR and BOS before clinical diagnosis. miRNAs were differentially expressed in exosomes from BOS and AR LTxRs compared with stable LTxRs.
Gunasekaran, 2018 [151]	10 LTxRs diagnosed with BOS, and 10 stable LTxRs	Sample type: serum Analysis: Western blot, using antibodies to lung SAg (K-a-1-tubulin [Ka1T] and collagen V [Col-V]), MHC class II molecules, costimulatory molecules CD40, CD80, and CD86, and transcription factors class II MHC trans-activator, NF-kB, hypoxia-inducible factor 1-a, IL-1R–associated kinase 1, MyD88, and 20S proteasome C57BL/6 mice immunized with sEV from BOS but not stable LTxR	Lung SAg (K-a-1-tubulin [Ka1T] and collagen V [Col-V]), MHC class II molecules, co-stimulatory molecules CD40, CD80, and CD86, and transcription factors class II MHC trans-activator, NF-kB, hypoxia-inducible factor 1-a, IL-1R–associated kinase 1, MyD88, and 20S proteasome were detected in exosomes from BOS, but not in stable LTxR. In contrast, adhesion molecules were present in both groups. C57BL/6 mice immunized with exosomes from BOS but not stable LTxR demonstrated Ab to SAg and HLA.
Mohanakumar, 2019 [152]	90 bilateral LTxRs included: 5 with PGD grade 3, 5 with PGD grade 0, 15 with symptomatic RVIs requiring intervention, 15 LTxRs without RVI and in stable condition, 10 AR, 10 without AR, 5 with de novo DSA, 5 without DSA, 10 diagnosed with BOS, and 10 stable LTxRs	Sample type: serum Analysis: Western blot, using antibodies to lung self-antigens K alpha 1 tubulin and collagen-V, costimulatory molecules (CD80, CD86), transcription factors (nuclear factor kappa-light-chain-enhancer of activated B cells, hypoxia-inducible factor 1a, Class II MHC Transactivator), and 20S proteasome	LTxRs with grade 3 PGD, RVI, AR, and DSA had exosomes containing SAgs; exosomes from stable recipients did not. LTxRs with grade 3 PGD, AR, and DSA had exosomes containing also costimulatory molecule 80, costimulatory molecule 86, MHC class II and 20S proteasome.
Bansal, 2020 [153]	90 bilateral LTxRs included: 3 diagnosed with BOS, 3 with AR, 3 with symptomatic RVIs and 5 stable LTxRs	Sample type: serum Analysis: LC−MS/MS, Western blot to validate the signatures identified by mass spectrometry	2 unique proteins for AR, 4 for RVI, 24 for BOS, and 8 for stable LTxRs. Differential analysis of AR, BOS, RVI, and stable patients identified significantly deregulated proteins in each condition (31, 2, and 2, respectively). Exosomes from LTxRs with AR contained proteins involved in immunoglobulin, complement regulation, coagulation, and innate and adaptive immune response pathways. Exosomes from LTxRs with BOS revealed enriched immunoglobulin receptors and a carboxypeptidase N catalytic chain. Exosomes from LTxRs with RVI had an enriched macrophage-stimulating factor.
Sharma, 2020 [154]	LTxRs at 2 transplant centers 41 with BOS 30 stable time-matched controls	Sample type: serum at diagnosis of BOS and at 6 and 12 months before the diagnosis Analysis: Western blot, using antibodies to lung self-antigens K alpha 1 tubulin and collagen-V	Exosomes from LTxRs with BOS (*n* = 21) showed increased levels of lung Sags compared with stable LTxRs (*n* = 10). A validation study using 2 separate cohorts of LTxRs with BOS and stable time-matched controls from 2 centers also demonstrated significantly increased lung SAgs−containing exosomes at 6 and 12 months before BOS.
Sharma, 2020 [155]	19 pediatric LTxRs 6 with BOS 13 stable time-matched controls	Sample type: serum at diagnosis of BOS and at 6 and 12 months before the diagnosis Analysis: Western blot, using antibodies lung self-antigens, co-stimulatory molecules transcription factors, major histocompatibility complex class II, adhesion molecules, and 20S proteasome	Circulating exosomes from BOS LTxRs contained increased levels of SAgs, donor HLA class I, MHC-II, transcription factors, co-stimulatory molecules, and 20S proteasome compared with stable. LTxRs. Serial analysis of exosomes containing SAgs demonstrated that exosomes are detectable in the circulation before BOS.
Gunasekaran, 2020 [156]	35 LTxRs with symptomatic lower- and upper-tract RVIs, and 32 stable LTxRs	Sample type: serum at diagnosis of RVI Analysis: Western blot, using antibodies lung self-antigens, 20S proteasome and viral antigens for rhinovirus, coronavirus, and respiratory syncytial virus	Exosomes from LTx Rs diagnosed with RVIs contained lung self-antigens, viral antigens, and 20S proteasome and elicited immune responses to lung self-antigens that resulted in development of chronic lung allograft dysfunction in immunized mice.
Itabashi, 2020 [157]	18 LTxRs with BOS 34 stable LTxRs	Sample type: plasma and BAL fluid samples were collected from patients 2 to 3 years after LTx Analysis: sEV from BAL fluid or plasma were analyzed for SAgs, natural killer cells markers, and cytotoxic molecules. Western blot with Abs specific to target proteins, including Col-V, CIITA, NF-κB, Kα1T, 20S proteasome, CD56, NKG2D (sc-23869), perforin (sc-373943), FasL	Exosomes derived from BAL fluid of LTxRs with BOS (compared with BAL fluid from stable LTxRs) had elevated levels of lung SAgs Col-V, Kα1T, CIITA, NF-κB, and 20S proteasome, showed also higher levels of the NK-cell-associated molecules CD56 and NKG2D (compared to stable LTxRs), and also demonstrated the presence of cytotoxic molecules: perforin, FasL. Exosomes from plasma samples of LTxRs with BOS also contained NK cells associated (CD56, NKG2D) and cytotoxic molecules Perforin, FasL as noted in BAL but not in stable and healthy volunteers.
Goodlet, 2020 [158]	1 LTx Rs with TCMR and SARS-CoV2-infection	Sample type: serum Analysis: Western blot, using antibodies to collagen V [COL V] and Kα1 tubulin [Kα1T]), HLA-DQ and HLA-DR, and SARS-CoV-2 spike protein	During rejection, but before SARS-CoV-2 infection, lung self-antigens and HLA class II molecules were found in exosomes. After the diagnosis of COVID-19, exosomes with the SARS-CoV-2 spike protein were also found. After the resolution of infectious symptoms, exosomes with SARS-CoV-2 spike protein were no longer detected; however, exosomes with lung self-antigens and HLA class II molecules persisted, which coincided with a progressive decline in spirometric flows, suggesting chronic lung allograft dysfunction.
**Heart**			
Sukma Dewi, 2017 [159]	10 HTx Rs including 5 with AR 5 without AR	Sample type: serum Analysis: qPCR-based microRNA-profiling assay for 175 microRNAs	Exosomal miR-142-3p is increased during cardiac allograft rejection and augments vascular permeability through down-regulation of endothelial RAB11FIP2 expression
Kennel, 2018 [160]	10 healthy controls; 10 HF patients without allograft; 10 HTx Rs without rejection; 10 HTx Rs with TCMR 8 HTx Rs with ABMR	Sample type: serum Analysis: LC-MS/MS	45 EV proteins distinguished between 3 groups: (1) control and heart failure (HF); (2) HTx without rejection; and (3) TCMR and ABMR. 15 EV proteins were differentially expressed in non-rejection HTx, TCMR and ABMR. 8 out of these 15 proteins play a role in immune response. The majority of identified proteins were associated with complement activation, adaptive immunity such as immunoglobulin components and coagulation.
Hu, 2020 [161]	4 HTx Rs including 1 with ABMR	Sample type: serum collected up to 26 days of perioperative follow-up Analysis: exosomes subset specific to donor HLA I; Western blot using antibodies to troponin T, cytochrome c, flotillin 1, and C4d; mRNA analysis	Exosomes showed expression of troponin protein and mRNA at all follow-up time points. In 1 HTxR who developed ABMR on day 14 endomyocardial biopsy time-specific C4d protein was present in a donor heart exosome subset, which resolved with the treatment. C4d was not seen in other 3 patients’ donor exosomes.
Castellani, 2020 [162]	90 HTx Rs including 53 training cohort (based on endomyocardial) biopsy result including 33 with no rejection 11 TCMR (grade 2R or 3A) 7 ABMR (grade 1 or 2 in + positive DSAs) 37 validation cohort (regardless of the final histologic diagnosis)	Sample type: serum Analysis: sEV surface marker analysis by multiplex flow cytometry	The concentration of exosomes was significantly increased and their diameter decreased in Rs with both TCMR and ABMR. Among exosomes surface markers, CD3, CD2, ROR1, SSEA-4, human leukocyte antigen (HLA)-I, and CD41b discriminated between controls and ACR, whereas HLA-II, CD326, CD19, CD25, CD20, ROR1, SSEA-4, HLA-I, and CD41b discriminated controls from ABMR.
**Kidney**			
Alvarez, 2013 [163]	15 KTx Rs	Sample type: urine Analysis: Western blot	NGAL expression in exosomes remained elevated in the patients with DGF compared with non-DGF patients.
Qamri, 2014 [164]	213 KTxRs + 14 KPTxRs Group 1 —ESKD secondary to diabetic nephropathy because of diabetes mellitus type I, who received a simultaneous kidney/pancreas transplant; Group 2—ESKD secondary to diabetic nephropathy (diabetes mellitus type I or type II), who received kidney allograft only; Group 3—ESKD secondary to congenital causes or acquired obstructive nephropathy; Group 4 —ESKD secondary to immune-complex mediated glomerulonephritides (IgA nephropathy, membranous glomerulonephritis, lupus nephritis); and Group 5 —unknown/unclassified ESKD 60 healthy donors prior to donation	Sample type: serum Analysis: sEV identified as CD31+/CD42b− microparticles and quantified by fluorescence-activated cell scanning	No differences in the quantity of circulating exosomes in the pre-KPTx or KTx recipient sera and healthy donor sera. Patients with ESKD secondary to diabetes mellitus, obstructive/inherited kidney disease and autoimmune disease had a decrease in both circulating exosomes and SCr by day 60 after KTx. An increase in circulating exosomes levels associated with AR, whereas in patients with no rejection the levels of circulating exosomes did not change; after treatment for rejection, circulating exosomes were rapidly decreased in patients with negative PTC C4d, but decrease was slower in patients with positive PTC C4d.
Peake, 2014 [165]	14 KTx Rs 11 healthy controls 9 CKD pts	Sample type: urine 4, 24 and 168 h after KTx Analysis: Expression of exosomal mRNA for the injury biomarkers neutrophil gelatinase-associated lipocalin (NGAL), interleukin-18 (IL-18), kidney injury molecule-1 (KIM-1), and cystatin C	Urinary NGAL and IL-18 levels reflect the day 7 creatinine reduction ratio (CRR). While mRNA for these biomarkers is present in exosomes, their levels do not reflect or predict urinary biomarker levels or the CRR. This likely reflects the fact that packaging of mRNA in exosomes is selective, and is not necessarily representative of mRNA in the parent cells responsible for biomarker production.
Sigdel, 2015 [166]	30 KTx Rs, including 20 with no AR 10 with AR	Sample type: urine Analysis: LC-MS/MS	Eleven urine exosomal proteins, functionally involved in an inflammatory and stress response, were more abundant in urine samples from patients with AR, three of which are exclusive for the exosomal fraction. Exosomal AR-specific biomarkers were also detected in unfractionated whole urine, but were observed at significantly lower abundance and were not significant for AR in unfractionated whole urine.
Park, 2017 [167]	30 KTx Rs, including 15 with no AR 15 with AR 3 BKV nephropathy 3 chronic AMR Validation cohort 14 KTx Rs, including 7 with no AR 7 with AR	Sample type: urine Analysis: a urine-based platform to detect kidney transplant rejection termed iKEA (integrated kidney exosome analysis)	Significantly higher level of CD3+ exosomes among patients undergoing cellular rejection, very low CD3+ EV levels in BKV (BK virus) nephropathy and chronic ABMR patients, which confirmed the specificity of iKEA to the TCMR.
Tower, 2017 [168]	93 KTx Rs with allograft dysfunction 23 healthy controls	Sample type: plasma Analysis: flow cytometry	In the 28 subjects with ABMR, the density of C4d+/CD144+ microvesicles was 11-fold higher than KTx Rs with no ABMR and 24-fold than for healthy volunteers. Densities of C4d+ and C4d+/annexin V+ (C4d+/AVB+) microvesicles were increased in ABMR compared with no ABMR and healthy subjects. C4d+/AVB+ microvesicles correlated with ABMR biopsy severity, C4d+/CD144+ microvesicle concentration decreased by 72% after the treatment.
Zhang, 2017 [54]	64 Ktx Rs including: 18 ABMR, 8 TCMR, 38 no rejection	Sample type: plasma Analysis: mRNA expression	Among 21 candidate genes, multiple genes were identified (gp130, CCL4, TNFα, SH2D1B, CAV1, atypical chemokine receptor 1 [Duffy blood group]) whose mRNA transcript levels in plasma exosomes significantly increased among ABMR compared with TCMR and/or control patients. A gene combination score calculated from 4 genes of gp130, SH2D1B, TNFα, and CCL4 was significantly higher in ABMR than TCMR and no rejection control groups.
Lim, 2018 [53]	47 KTx Rs 22 stable kidney function 25 TCMR	Sample type: urine Analysis: nano-UPLC-MS/MS, Western blot	17 proteins were increased in TCMR patients. Of all candidate biomarkers, tetraspanin-1 and hemopexin were significantly higher in TCMR patients.
Wang, 2019 [169]	9 KTx Rs from DCD donors	Sample type: serum Analysis type: miRNA expression profiling with high-throughput sequencing	Three co-expressed miRNAs, hsa-miR-33a-5p_R-1, hsa-miR-98-5p, and hsa-miR-151a-5p, were significantly upregulated in DGF.
Yang, 2019 [170]	42 KTxRs 28 with IF/TA, including 14 with ABMR and 14 patients with non-ABMR	Sample type: serum Analysis type: exosomal biomarkers detection by flow cytometry: (1) anti-CD63, anti-CD4, anti-CXCR5, anti-CXCR3, and anti-CCR6 (2) anti-CD63, anti-CD4, anti-CXCR5, anti-HLA-G, and anti-CTLA-4	No significant differences in CD4+CXCR5+ and CD4+CXCR5+CXCR3+CCR6-exosomes between ABMR and non-ABMR groups was detected, whereas the proportion of CD4+CXCR5+CXCR3-exosomes was significantly higher in ABMR group than that in non-ABMR group; CTLA-4 expression of CD4+CXCR5+exosomes was significantly lower in ABMR group than that in non-ABMR group. HLA-G expression was not significantly different between the groups. Tfh cell-derived exosomes in ABMR patients significantly promoted B cell proliferation and differentiation, compared with non-ABMR group, the percentage of B cells and plasma cells increased by 87.52% and 110.2%, respectively.
Saejong, 2020 [171]	KTx recipients Stable (*n* = 14) IF/TA I (*n* = 15) IF/TA II (*n* = 17) IF/TA III (*n* = 6)	Sample type: serum, kidney allograft biopsy (protocol biopsies) Analysis: Western blot with antibody against sEV proteins including anti-CD9, anti-CD63 Reverse Transcription Polymerase Chain Reaction (RT-PCR) and Real-time PCR miR-21, miR-142-3p, miR-221, RNU-44 (house-keeping miR for renal tissue) and cel-39.	Expression of miR-21, miR-142-3p and miR-221 in renal histology with high fibrosis score (Banff classification) was higher than in samples with a lower score (*n* = 17/group). However, expression of these miRs from plasma exosomes or from whole plasma of post-KT patients with different severity of IF/TA, as determined by percentage of IF/TA including: grade I (5–25%) (*n* = 15), grade II (26–50%) (*n* = 15), grade III (≥ 50%) (*n* = 6), versus stable graft function (no IF/TA) (*n* = 15) was not different. Nevertheless, high expression of miR-21 in exosomes, but not from whole plasma, was demonstrated in IF/TA grade II and III compared with IF/TA grade I.
Carreras-Planella, 2020 [172]	23 KTx Rs 7 normal kidney function, 5 IF/TA, 6 TCMR 5 calcineurin inhibitors toxicity 41 KTx Rs–validation cohort	Sample type: urine Analysis: LC−MS/MS, the results related to vitronectin were further validated with a preliminary ELISA assay in urine samples from a limited number of kidney-transplanted patients with different grades of fibrosis.	Differential expression of several proteins in urinary EVs among different groups of KTx Rs. Differential expression of vitronectin (VTN) in patients displaying chronic interstitial and tubular lesions (ci and ct mean > 2 according to Banff criteria).
Carreras-Planella, 2020 [173]	7 normal kidney function, 5 interstitial fibrosis and tubular atrophy, 5 calcineurin inhibitors toxicity	Sample type: urine Analysis: LC−MS/MS	Several proteins of the uroplakin family (UPK1A, UPK1B, UPK2, and UPK3A), as well as envoplakin (EVPL) and periplakin (PPL) (citolinker proteins) were significantly upregulated in urinary EVs in calcineurin inhibitors toxicity compared to IFTA and normal kidney function.
Takada, 2020 [120]	KTx patients (samples collected at the time of the protocol biopsy, and samples at the episode biopsy were excluded) including: 20 normal histology 19 IF/TA 17 calcineurin inhibitors toxicity 22 chronic active ABMR	Sample type: urine in all patients, frozen or paraffin sections of transplanted kidney biopsies Analysis type: exosomes-Western blot with antibody against SYT17 biopsies -immunohistochemistry with anti-SYT17, anti-STAT3 pY705, anti-phospho NFκB p65 Ser276 antibodies	No SYT17 protein was detected in whole-urine samples, SYT17 proteins were detectable in urinary exosomal fractions, high enrichment of SYT17 in exosomes from urine of chronic active AMR patients compared to healthy volunteers and individuals in the normal histology KTx. SYT17 protein was expressed strongly in the chronic active ABMR group compared to other KTx groups; SYT17 was mainly expressed in the tubular cells of the kidney but not in other cell populations including endothelial cells (glomeruli) and epithelial cells.
Freitas, 2020 [174]	23 KTx Rs (1st KTx)	Sample type: urine at 1 week, 1 month and 3 months post KTx Analysis type: miRNAs’ expression	Three overexpressed urinary exo-miRs (miR-146b, miR-155, andmiR-200a) in KTxRs were negatively correlated with TAC dose. miR-200a was positively correlated with proteinuria.
El Fekih, 2021 [121]	175 KTx Rs undergoing for cause biopsy >> 192 urine samples that have matched biopsy specimens were included	Sample type: urine, kidney allograft biopsy (for cause biopsies) Analysis type: RT-PCR and Real-time PCR for gene expression analysis	An exosomal mRNA signature discriminated between biopsy samples from patients with all-cause rejection and those with no rejection, an additional gene signature discriminated patients with TCMR from those with ABMR.
Chen, 2020 [175]	58 KTx Rs 27 healthy controls	Sample type: plasma at months 3, 6 and 12 Analysis type: miRNAs’ expression	Exosomal miR-21, miR-210 and miR-4639 showed negative correlations with eGFR in the training set and were selected for further analysis. In the validation set, miR-21, miR-210 and miR-4639 showed the capability to discriminate between subjects with chronic allograft dysfunction (eGFR < 60 mL/min/1.73 m^2^) and those with normal graft function.
**Pancreatic islets**			
Vallabhajosyula, 2017 [147]	5 ITx Rs followed up for 5 years 5 living donor KTx Rs	Sample type: plasma and/or urine Analysis type: affinity antibody-coupled bead purification of tissue-specific exosomes, western blot, RNA microarray	ITx: decrease in transplant islet exosome signal temporally correlated with recurrence of islet autoimmunity, which preceded the clinical onset of hyperglycemia. RNA cargo analysis showed expression of insulin, glucagon, somatostatin, and FXYD2, which was undetectable in the pre-transplant sample. KTx: post-transplant recipient plasma samples showed donor kidney–specific HLA-A2 and HLA-B27–positive exosomes; Western blot analysis of post-transplant plasma HLA-A2–bound exosomes confirmed expression of the renal epithelial protein aquaporin 2; post-transplant urine donor HLA-A2 exosomes showed the presence of the renal glomerular protein podocalyxin-1.
Korutla, 2019 [176]	ITx–case report	Sample type: serum Analysis type: affinity antibody-coupled bead purification of tissue-specific exosomes, western blot, RT-PCR	Decrease in transplant islet exosome signal temporally correlated with recurrence of islet autoimmunity, which preceded the clinical onset of hyperglycemia; analysis of purified transplant islet exosome cargoes showed decrease in insulin-containing exosomes, but not glucagon containing exosomes, together with time-specific increase in islet autoantigen, glutamic acid decarboxylase 65 (GAD65) indicating selective destruction of transplanted β cells secondary to recurrent T1D autoimmunity.
**Liver**			
Zhang, 2019 [177]	exosome analysis in 3 sets of paired patients, with and without AR proteomic analysis of liver biopsy 73 OLTx Rs with AR 63 OLTx Rs without AR	Sample type: serum, liver allograft biopsy (for cause biopsies) Analysis type: western blot with antibody against galectin-9	The rejection group showed significantly higher levels of galectin-9 in exosomes and galectin-9 expression in the livers.

AR—acute rejection, ABMR—antibody mediated rejection, BAL—broncho-alveolar lavage, BKV—BK virus, BOS—bronchiolitis obliterans syndrome, CAV—coronary artery vasculopathy, DGF—delayed graft function, DSA—donor specific antibodies, ESKD—end-stage kidney disease, HTx—heart transplant, IF/TA—interstitial fibrosis and tubular atrophy, ITx—islet transplantation, KTx—kidney transplant, KPTx—kidney/pancreas transplant, LC-MS/MS—liquid chromatography−tandem mass spectrometry, LTx—lung transplant, MHC—major histocompatibility complex, nano-UPLC-MS/MS—nano-ultra performance liquid chromatography-tandem mass spectrometry, OLTx—orthotopic liver transplantation, PGD—primary graft dysfunction, Rs—recipients, RVI—respiratory viral infection, SAgs—selfantigens, TCMR—T-cell mediated rejection, TG—transplant glomerulopathy.

## Data Availability

Not applicable.
